# DNA Intercalators Inhibit Eukaryotic Ribosomal RNA Synthesis by Impairing the Initiation of Transcription

**DOI:** 10.3390/genes12091412

**Published:** 2021-09-14

**Authors:** William J. Andrews, Swagat Ray, Tatiana Panova, Christoph Engel, Konstantin I. Panov

**Affiliations:** 1 School of Biological Sciences, Queen’s University Belfast, Belfast BT9 5DL, UK; wandrews@belfastmet.ac.uk (W.J.A.); swaray@lincoln.ac.uk (S.R.); t.panova@qub.ac.uk (T.P.); 2 School of Life Sciences, University of Lincoln, Lincoln LN6 7TS, UK; 3 Regensburg Center for Biochemistry, University of Regensburg, 93053 Regensburg, Germany; christoph.engel@ur.de; 4 Patrick G Johnston Centre for Cancer Research, Queen’s University Belfast, Belfast BT9 5DL, UK

**Keywords:** DNA intercalators, ribosome biogenesis, ribosomal RNA, RNA polymerase I, ribosomal DNA, transcription

## Abstract

In eukaryotes, ribosome biogenesis is driven by the synthesis of the ribosomal RNA (rRNA) by RNA polymerase I (Pol-I) and is tightly linked to cell growth and proliferation. The 3D-structure of the rDNA promoter plays an important, yet not fully understood role in regulating rRNA synthesis. We hypothesized that DNA intercalators/groove binders could affect this structure and disrupt rRNA transcription. To test this hypothesis, we investigated the effect of a number of compounds on Pol-I transcription in vitro and in cells. We find that intercalators/groove binders are potent inhibitors of Pol-I specific transcription both in vitro and in cells, regardless of their specificity and the strength of its interaction with DNA. Importantly, the synthetic ability of Pol-I is unaffected, suggesting that these compounds are not targeting post-initiating events. Notably, the tested compounds have limited effect on transcription by Pol-II and III, demonstrating the hypersensitivity of Pol-I transcription. We propose that stability of pre-initiation complex and initiation are affected as result of altered 3D architecture of the rDNA promoter, which is well in line with the recently reported importance of biophysical rDNA promoter properties on initiation complex formation in the yeast system.

## 1. Introduction

In eukaryotes, ribosome biogenesis (RiBi) is a fundamental cellular process in which all three nuclear polymerases are involved. Pol-I transcribes rRNA as a 47S-rRNA precursor which is processed into mature 28S, 18S and 5.8S rRNAs. Pol-II transcribes all mRNAs including those that encode ribosomal proteins, and Pol-III transcribes the 5S rRNA, tRNAs and other small RNAs. Aberrations of RiBi that allow an increased level of ribosome production and consequently an increased rate of protein synthesis which supports cell growth are commonly found in aggressive cancers [1]. Pol-I transcription is seen by many as the key regulator of RiBi, and any changes in Pol-I transcriptional activity result in dramatic effects on the cell [2,3].

**Figure 1:**
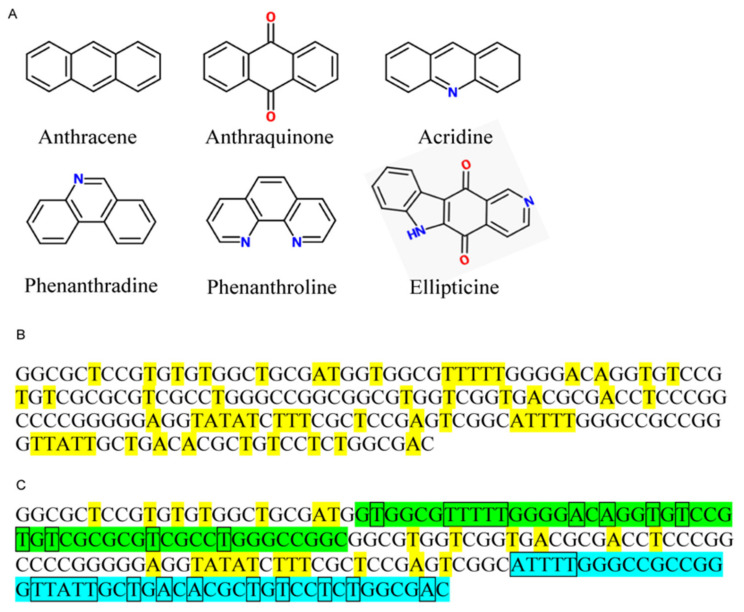
Structural precursors to common DNA intercalating compounds and the sequence arrangement of the rDNA promoter region. (**A**) All currently available intercalating compounds derive from just six basic chemical structures. (**B**) Sequence of the human Pol-I promoter with all adenine and thymine residues highlighted (yellow). (**C**) Sequence of the human Pol-I promoter with the UCE (green) and core promoter (blue) are highlighted, with adenine and thymine residues within these regions boxed.

Many DNA binding proteins and drugs bind to DNA through interactions with the minor and major grooves, and compounds that can bind into the grooves or compete out pre-bound proteins are capable of inhibiting a variety of cellular processes [4]. Although there are currently hundreds, if not thousands of known DNA intercalators available, almost all structures derive from six basic compound classes: ellipticines, acridines, anthraquinones, anthracenes, phenanthridines and phenanthrolines (Figure 1A). For therapeutic use, however, there are only four organic intercalators approved for use by the Food and Drug Association, USA (FDA), Doxorubicin and Daunorubicin (anthracyclines), Mitoxantrone (an anthracenedione) and Amsacrine (an aminoacridine), and they all function by insertion into the minor groove [5,6,7]. 

In recent years it has become evident that selective inhibition of Pol-I transcription has a potential to become a novel and efficient therapeutic approach in the fight against cancer. Pioneering work by the Hannan group has convincingly demonstrated a high sensitivity of cancer cells to inhibition of Pol-I transcription [3]. However, only a few small compounds inhibiting Pol-I transcription are known and only one has progressed to Phase II of clinical trials [8] (Trial code: NCT02719977). Therefore, development of new compounds targeting Pol-I transcription is essential for any progress in this area. We have previously demonstrated that the alkaloid 9-Hydroxyellipticine (9HE), initially believed to function through inhibition of Topoisomerase IIα (Top2α), is also functioning by intercalation into the GC rich regions of the rDNA promoter, leading to displacement of Pol-I basal transcription factor SL1 from the promoter and subsequently, Pol-I inhibition [9]. A similar mechanism of action was also assigned to the clinically relevant Pol-I inhibitor CX-5461 [8], although a recent study suggests that the compound could also inhibit Pol-I promoter escape [10]. TATA binding protein (TBP)-containing complex SL1 (TIF-IB) is responsible for promoter recognition and Pol-I recruitment and is essential for rRNA transcription [11,12].

The Pol-I promoter is predominantly composed of guanine and cytosine residues and we can expect that GC-intercalators may efficiently inhibit transcription (similar to ellipticines). However, the promoter also contains four stretches of five AT nucleotides (Figure 1B, upper panel) which suggests that AT- intercalators may also have an inhibitory effect. Further examination of the promoter sequence reveals that three of these AT pentanucleotide stretches reside in the upstream control element (UCE) (one AT stretch) and the core (two AT stretches) promoter regions (Figure 1B, lower panel). The UCE and core regions are the binding sites for upstream binding factor (UBF) and SL1 respectively. It is therefore also possible that AT intercalating agents could disrupt the association of these factors with the rRNA promoter.

In this study we show that DNA intercalators with GC, AT or general (AT or GC) DNA binding affinity can inhibit Pol-I transcription both in vitro and in cells without having a significant effect on general Pol-II/III transcription. Notably, effect on Pol-I transcription can be detected relatively earlier (30 min after treatment), whereas longer incubation leads to downregulation of Pol-II/III transcription. Importantly, we found that these compounds act predominantly by impairing recruitment of Pol-I to the rRNA promoter without affecting the synthetic ability of Pol-I. We also found that intercalating agent, and topoisomerase I (Top 1) inhibitor camptothecin (CPT) inhibits Pol-I transcription in vitro and its inhibitory activity is not Top 1 mediated.

## 2. Materials and Methods

### 2.1. Compounds

Camptothecin (CPT), methyl green (MG), crystal violet (CV), propidium iodide (PI), ethidium bromide (EtBr or EB), proflavine (Pro), 4′,6-diamidino-2-phenylindole (DAPI), thionine (Thio), acridine orange (AO) and bisBenzamide (Hoechst 33258) were of analytical grade, purchased from Sigma Aldrich (St. Louis, MO, USA) and reconstituted as 10 mg/mL stock solutions in ddH_2_0 and stored at −20 °C. CX-5461 was a kind gift from Ross Hannan (ANU).

### 2.2. Tissue Culture

HCT116 p53^+/+^ and ^−/−^cells were maintained in recommended McCoy’s 5A media (Gibco, Waltham, MA, USA) in a 37 °C, 5% CO_2_ incubator. The culture media was supplemented with 10% FBS (PAA) and 10,000 U/mL penicillin and 10,000 µg/mL streptomycin (Gibco, Waltham, MA, USA). Drug treatments were administered as indicated in figure legends at the concentrations stated.

### 2.3. Antibodies

All antibodies are described in Supplementary Table S1.

### 2.4. Plasmids, DNA Fragments and Primers

All primers and probes are described in Supplementary Table S2. The pcfTAF110 plasmid sequence is shown in Supplementary Data S1, fragment CMV-Fr sequence is shown in Supplementary Data S2.

### 2.5. In Vitro Transcription Assays

Non-specific Pol I driven transcription assays were performed as described [13] and samples were analyzed using a Tri-Carb 1600 TR-Liquid Scintillation Counter analyzer set to 50 s per read. 

Specific Pol I driven transcription reactions were performed as described [14,15] using 200 ng of supercoiled plasmid DNA or immobilized rDNA promoter. Reactions were supplemented with 1 µL HeLa nuclear extract or with highly purified general transcription factors (GTF: 2 µL SL1, 2.5 µL Pol-I and 0.1 µL UBF). S1 nuclease protection was performed as described [14] with a 60 bp ^32^P-5′-end labelled oligonucleotide containing a sequence identical to the region between −20 and +40 of the template strand in the human rRNA gene promoter. The amount of RNA produced by in vitro transcription reactions was quantified with the aid of a phosphorImager, FLA-7000 (Fuji) and Aida software (version 4.15, Raytek). The standard deviations were calculated from three independent experiments. 

Analysis of the specific Pol-II driven transcription was performed using the approach described in [16] with modifications described below.

The pcfTAF110 plasmid (Supplementary Data S1), a derivative of pcDNA3.2/V5-DEST (Invitrogen, Waltham, MA, USA) was used as a template for the amplification of the 1354 bp linear DNA fragment CMV-Fr (contains CMV promoter and flanking sequences, see Supplementary Data S2). The 5’-biotynilated DNA template_Pol_II_F and non-modified template_Pol_II_R primers (Supplementary Table S2) were used to obtain 5’-biotynylated DNA fragment.

Amplified DNA fragment was purified using the PureLink™ PCR Purification Kit (Invitrogen) and immobilized onto Dynabeads M280 magnetic beads (Invitrogen). Bead immobilization was performed as recommended by the manufacturer (10 µg of dsDNA per 100 µL beads) and beads stored at 4 °C in the binding buffer. The binding efficacy of about 90% resulted in a DNA concentration of 90 ng/µL. Prior to transcription reaction setup, beads were washed three times in the transcription buffer and then transferred into new tubes. Typically, 5 µL beads (450 ng DNA) was used per transcription reaction.

The transcription reaction contains 5 μL HeLa nuclear extract, 450 ng of immobilized DNA template in the transcription buffer (20 mM HEPES, pH 7.9, 100 mM KCl, 3mM Mg Cl_2_, 0.2 mM EDTA, 0.5 mM DTT, 20% glycerol) and 40U RNasin (Fermentas). Reactions were supplemented with various compounds or vehicle, mixed and incubated at 30 °C for 30 min. The transcription reaction was then initiated by adding 10 mM NTP mix (final concentration 400 µM each, except for the negative control where water was added, ‘noNTP control’) and maintained at 30 °C for 1 h. The transcription was terminated by addition 150 μL stop solution (125 mM Tris-HCl pH 7.5, 12.5 mM EDTA, 150 mM NaCl, 1% SDS) and immobilized template was removed using a magnetic rack. A total 700 μL of the RLT buffer from the Qiagen RNeasy Micro kit was added to each sample. RNA was purified as recommended by the manufacturer, including on-column DNase treatment but without the addition of carrier RNA. The RNA was eluted using 15 μL water and stored at −80 °C. A total 50 ng of free DNA template was subjected to the same purification procedure and used as a control for DNA removal efficiency, ‘DNA control’.

For reverse transcription with the Qiagen Sensiscript RT kit, 2 μL of the purified RNA and 1 μM Pol_IITr _R primer (Supplementary Table S2) were used in a final reaction volume of 20 μL. A total 2 mL of water was used for a negative ‘RT control’.

For quantitation the sequence-specific fluorescent labelled probe Pol-II_Pr (Supplementary Table S2) at 200 nM, Pol_IITr2_F and Pol_IITr2_R primers (Supplementary Table S2) at 400 nM each and QuantiNova PCR Kit (Qiagen, Hilden, Germany) were used. We used 2 μL of the reverse transcription reaction to set up qPCR reactions with a final volume of 10 μL in 384 well plate. Reactions were set in triplicates and following controls were used: Positive: reactions were supplemented with various amounts of nuclear extract; negative: non-template control, ‘no NTP control’, ‘DNA control’ and ‘RT control’. Two step qPCR was performed in LightCycler480 thermocycler (Roche, Basel, Switzerland) as recommended by PCR Kit manufacturer. qPCR standard curve was prepared from the CMV-Fr DNA fragment and used for RNA copy number calculations.

### 2.6. Nascent RNA Detection in Cells

Cells were grown until 50–60% confluent on sterilized glass slides (Superfrost^®^ Plus microscope slides, Menzel-Glaser) and then were treated with compounds or vehicle (as indicated) for 30 min, followed by the addition of 5EU (5-Ethynyl-uridine, 1 mM final concentration) and further incubation for 1 h. The following concentrations were used: Actinomycin D (ActD): 3 µM; ethidium bromide (EB): 40 µM; proflavine (Pro): 2 µM; CX5461: 5 µM.

Cells were fixed with 3.7% paraformaldehyde and permeabilized with 0.5% Triton X-100 in PBS. Nascent RNA was fluorescently labelled using Click-IT^®^ Cell Reaction Buffer Kit (Invitrogen, Waltham, MA, USA) supplemented with Cy5 Azide (Sigma Aldrich) according to manufacturer’s instruction. Nuclei were stained with Hoechst 33342 and images were acquired with a Leica TCS SP5 confocal microscope equipped with an x63 oil-filled objective. Leica Application Suite X (LAS X) software was used for quantification of 5EU incorporation. Box-and-whisker plots of quantification of 5EU incorporation were obtained with Excel. They show the median, the 25% and 75% quantiles, as well as outliers.

### 2.7. Analysis of Pol-I, Pol-II and Pol-III Transcription in Cells

Cells were grown until 70% confluent and then treated with compounds for 30 min. The following concentrations were used: ActD: AD1–3 nM; AD2–3 µM; Camptothecin (CPT): 8 µM; ethidium bromide (EB): 40 µM; crystal violet (CV): 20 µM; Pro: 2 µM; CX5461: 5 µM. RNA was purified using the RNeasy purification kit (Qiagen) following the manufacturer’s instructions and the RNA concentration was determined spectroscopically. A total 1 µg of RNA was converted to cDNA using the High Capacity RNA-to-cDNA reverse transcriptase kit (Applied Biosystems, Waltham, MA, USA). Then 47/45S pre-rRNA levels were analyzed on the LighCycler 480 thermocycler (Roche) using primers and conditions described previously [17]. 

Pol-II and Pol-III transcripts were analyzed as described previously [9]. All signals were normalized to 18S rRNA level. The standard deviation was calculated from three independent experiments.

### 2.8. Chromatin Immunoprecipitation Assay (ChIP)

ChIP assays were carried out as described previously [18]. In brief, cells were grown until 70% confluent, then treated with compounds for 30 min. The following concentrations were used: ethidium bromide (EB)—9.16 µM; crystal violet (CV)—5 µM; proflavine (Pro)—0.5 µM. Cells were cross-linked with formaldehyde (final concentration 1%) for 10 min, and the cross-linking stopped by the addition of glycine (final concentration 0.125 M) for 5 min. Cross-linked chromatin was sheared using a Biorupter (Diagenode, Liege, Belgium) until 300-base pair average size. Immunoprecipitation was carried out using chromatin isolated from 1 × 10^6^ cells, antibodies listed in Supplementary Table S2 and 30 μL of Protein A/Protein G magnetic beads (Invitrogen). Purified immunoprecipitated DNA was analyzed by two tetraplex quantitative PCR panels designed for eight regions of the rDNA repeat; promoter, intergenic spacer 4 (IGS4), 18S, 5.8S, 28S, terminator, IGS1, and 5′externally transcribed spacer (ETS). Reactions were carried out on a LightCycler 480 with a reaction volume of 10 μL per well in triplicate. Signal were quantified using internal standards and specific signals were calculated as a difference between the signals from the specific antibody and from the negative control (an appropriate IgG). Results were expressed as the amount relative to input chromatin and normalized to control IgG levels, with the standard deviations calculated from three independent ChIP experiments.

## 3. Results

### 3.1. Intercalators and Groove Binders Inhibit Pol-I Transcription Initiation

We tested a range of DNA intercalators and minor/major groove binders for the potential to inhibit Pol-I and determined IC_50_ for each drug using an in vitro Pol-I specific transcription assay. This assay primarily measures the efficiency of the initial stages of transcription which include preinitiation complex (PIC) formation, first phosphodiester bond synthesis and promoter clearance (see reviews [19] and for a detailed description of the Pol-I transcription cycle). The reactions were supplemented with supercoiled rDNA template and HeLa nuclear extract (NE) and the level of specific transcript was determined by S1 nuclease protection assay as described [14]. The results are summarized in Table 1. Interestingly, many of the compounds tested are very efficient inhibitors of specific transcription with IC_50_ at the nanomolar and micromolar range.

We found no apparent correlation between the specificity of the DNA binding of the compound and its inhibitory activity (Table 1) but observed a weak correlation (Pearson’s coefficient = 0.33) between the strength of the compound-DNA interactions (1/K_D_, see Table 1) and its inhibitory activity. Interestingly, ethidium bromide (a phenanthradine derivative; EtBr), a well-known mutagen, was the most potent inhibitor of Pol-I transcription in vitro (IC_50_ 61.8 nM). 

Next, we determined IC_50_ concentrations for EtBr, Pro, CPT, DAPI and CV in cells by measuring the level of Pol-I transcription at various compound concentrations (Table 2). For this assay we used the HCT116 p53^−/−^ cell line to avoid any effect of p53 dependent repression of Pol-I transcription. Notably, we observed a strong correlation between the IC_50_ determined in cell based and in vitro assays (compare Table 1; Table 2). Surprisingly, EtBr was still able to inhibit transcription (IC_50_ 9.16 µM), even though it has been reported that EtBr is actively excluded from viable cells [20].

**Figure 2:**
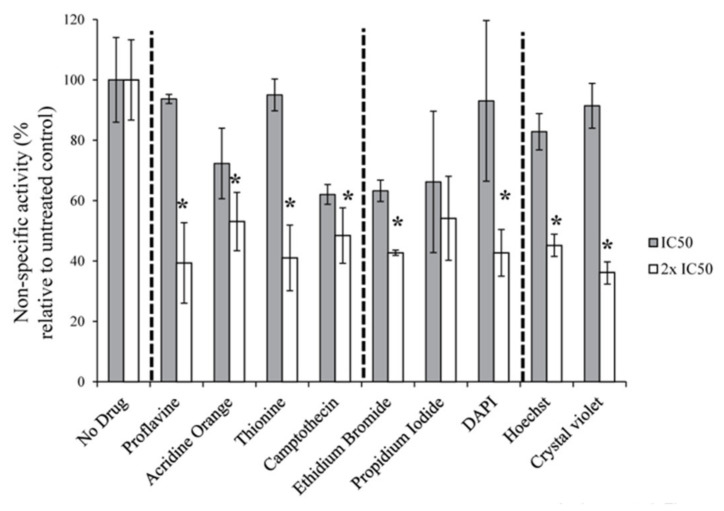
Pol-I synthetic activity is not affected by intercalators. 200 ng of calf thymus DNA was pre-incubated with either IC_50_ or 2 × IC_50_ (specific Pol-I activity, Table 1). Dashed lines separate untreated control samples (**left**), GC intercalating compounds (second from left), general DNA intercalating compounds (third from left) and AT intercalating compounds (**right**). Reactions were initiated by addition of HeLa Nuclear extract and after 30 min incubation the efficiency of non-specific RNA synthesis was determined using scintillator counter (in duplicates). The data expressed as a percentage of the highest value (set at 100%), and represents an average from two independent experiments (*n* = 2); standard deviations are shown; P-values were calculated using one and two-way ANOVA on R software, * *p* < 0.05. IC_50_—grey bars, 2 × IC_50_—white bars.

We also performed non-specific in vitro transcription assays which measure non-promoter driven transcription by Pol-I to determine the effect of intercalators and groove binders on the ability of Pol-I to synthesize RNA. We found that all compounds were unable to significantly affect Pol-I synthetic activity at the IC_50_ concentrations (obtained for promoter specific inhibition, Table 1) and only at double the IC_50_ concentrations detectable inhibition was observed (Figure 2).

These results imply that the catalytic activity of Pol-I is not the main target of the compounds and the inhibitory effect observed in the specific transcription assay cannot be fully linked to inhibition of Pol-I catalytic activity and phosphodiester bond synthesis.

### 3.2. DNA Intercalators/Groove Binders Have Limited Effect on Specific Pol-II Driven Transcription in Cell Free Assay

**Figure 3:**
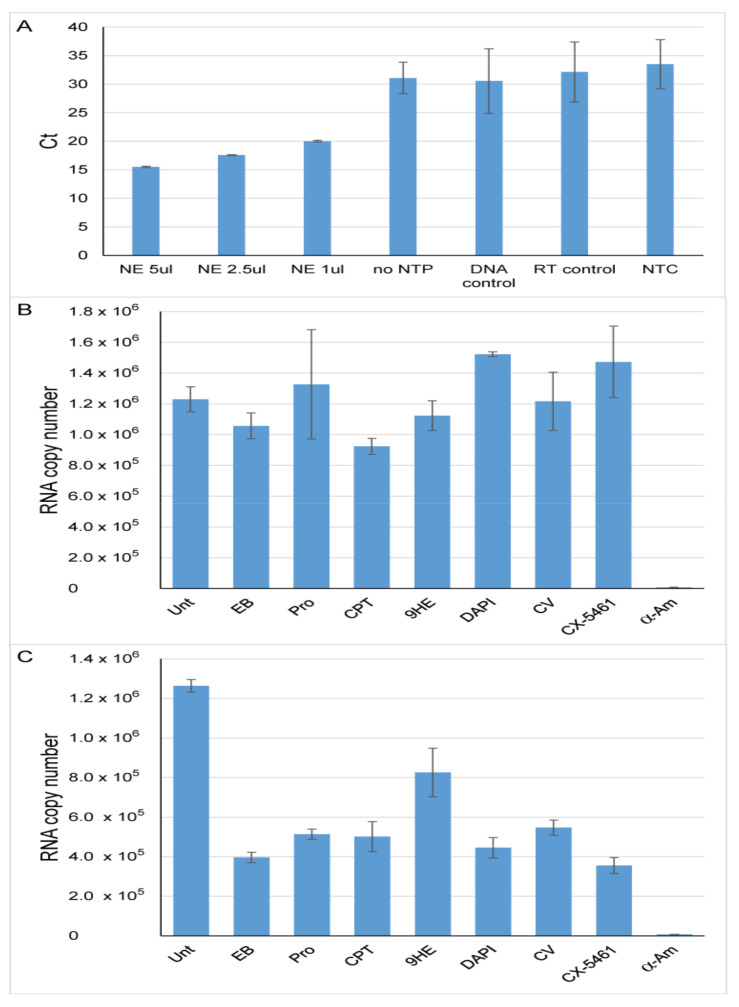
DNA intercalators/groove binders have limited effect on specific Pol-II driven transcription. In vitro transcription was performed and analyzed as described in Material and Methods. (**A**) Ct values for positive and negative controls were plotted in a bar graph. Standard deviation from three independent experiment are shown; *n* = 3. Positive controls: In vitro transcription reactions were supplemented with 5, 2.5 and 1 µL of HeLa NE as indicated. Negative controls: No NTP—transcription reaction was not supplemented with NTP’s; DNA control—45 ng non-immobilized DNA template (CMV_Fr) was subjected to the same treatment as transcription reactions including DNAse I treatment, column purification and reverse transcription; RT control—reverse transcription reaction was supplemented with water not RNA; NTC—no template control, a PCR reaction was supplanted with water; (**B**) Transcription reactions were supplemented with various compounds (at concentration equal to 2 × IC_50_ for Pol-*I* in vitro) or left untreated as indicated. The level of RNA synthesis is expressed as RNA copy number and plotted in a bar graph. Standard deviation from three independent experiments are shown; *n* = 3. Unt—no compound added; EB—124 nM ethidium bromide; Pro—700 nM proflavine; 9HE—840 nM 9-hydroxyellipticine; CPT—2.2 µM camptothecin; DAPI—1.32 µM 4′,6-diamidino-2-phenylindole; CV—69 µM crystal violet; CX-5461—240 nM CX-5461; α-Am—400 µM α-amanitin. (**C**) Transcription reactions were supplemented with various compounds (at concentration equal to 5 × IC_50_ for Pol-I in vitro) or left untreated as indicated. The level of RNA synthesis is expressed as RNA copy number and plotted in a bar graph. Standard deviation from three independent experiments are shown; *n* = 3. Unt—no compound added; EB—312 nM ethidium bromide; Pro—1.75 µM proflavine; 9HE—2.1 µM 9-hydroxyellipticine; CPT—5.5 µM camptothecin; DAPI—3.3 µM 4′,6-diamidino-2-phenylindole; CV—172 µM crystal violet; CX-5461—600 nM CX-5461; α-Am—400 µM α-amanitin.

All three nuclear polymerases (Pol-I, Pol-II and Pol-III) exhibit similar catalytic properties and share the same overall structure and mechanism of transcription [31,32,33,34]. The results of the non-specific assay suggest that intercalators do not affect the catalysis; however, these compounds may interfere with other stages of the transcription cycle (e.g., transcription bubble formation, first phosphodiester bond synthesis or a promoter escape) and this interference may be different for different enzymes. To test this, we analyzed the effect of compounds listed in Table 2, the specific inhibitor of Pol-I transcription CX-5461 that has no effect on Pol-II transcription [8]), and the concentration dependent inhibitor of Pol-II transcription α-amanitin on Pol-II specific transcription in vitro. We used a modified version of a non-radioactive eukaryotic in vitro transcription assay described previously [16]. Reactions were supplemented either with the compounds listed in Table 2 or with CX-5461 (IC_50_ determined in this work, Supplementary Table S3) or α-amanitin. For compounds listed in Table 2 and CX-5461 concentrations equal to × 2 IC_50_ and × 5 IC_50_ (Table 1 and Supplementary Table S3) were used, and α-amanitin was used at 400 µM, a concentration that inhibits both Pol-II and Pol-III activity [35]). Combinations of CX-5461 and compounds listed in Table 2 were also used (Figure 3). To ensure that accuracy and specificity of this PCR based assay we used a number of negative controls that include: ‘no NTP control’—transcription reaction was not supplemented with NTPs resulting in no RNA synthesis, ‘DNA control’—non-immobilized template (10% from immobilized template) was subjected to the same treatment as transcription reactions including DNAse I treatment and column purification, ‘RT control’—reverse transcription was supplemented with water not RNA and NTC—no template control, a PCR reaction was supplanted with water. Reactions supplemented with different amounts of HeLa nuclear extract were used as a positive control. Results are shown in Figure 3A, and demonstrate high Ct (cycle threshold) values in all negative controls and proportionality of Ct values to the amount of nuclear extract, confirming the specificity and accuracy of the assay.

To quantify the effect of various compounds on Pol-II transcription, an absolute quantification approach was used and a standard curve was generated using serial dilutions of the CMV-Fr DNA fragment. This allowed the results of these experiments to be expressed as the RNA copy number (Figure 3B,C). None of the compounds listed in Table 2 and CX-5461 had a significant effect on Pol-II transcription at a concentration equal to 2 × IC_50_. As expected, 400 μM α-amanitin completely repressed transcription by Pol-II (Figure 3C). At concentrations equal to 5 × IC_50_, we observe an inhibitory effect for all tested compounds (Figure 3). 

Together, these results revealed that the Pol-II promoter driven transcription in vitro is not significantly affected by the intercalators and grove-binders at concentrations that impairs Pol-I specific transcription, suggesting that Pol-I driven transcription is hypersensitive to these types of DNA binding agents.

### 3.3. DNA Intercalators/Groove Binders Have Limited Effect on General Transcription by Pol-II and Pol-III in Cells

Our results suggest that in vitro Pol-I promoter driven, but not non-specific transcription is hypersensitive to the intercalators and grove-binders. We also found that these compounds inhibit Pol-I transcription in cells (Table 2). Although we see no significant effect of the intercalators and grove-binders on specific Pol-II transcription in vitro we performed experiments aiming to explore effect of these compounds on general level of Pol-II/III transcription in cells. To this end we used two approaches. Firstly, we measure the level of nascent RNA synthesis in HCC116 p53^−/−^ cells treated with proflavine, ethidium bromide, CX-5461 (specific inhibitor of Pol-I transcription) at concentrations sufficient to inhibit about 90% of Pol-I transcription as shown in Figure 5A. As a control we used high concentration of ActD (3µM) that inhibits activity of all nucleolar polymerases [35]. Cells were treated with the selected compounds or a vehicle for 30 min and then 5EU (5-Ethynyl-uridine) was added and incubation was continued for another hour. During this time, 5EU is incorporated into any newly synthesized RNA, and the level of incorporation can later be assessed using a combination of “click” approach and confocal microscopy [36]. 

**Figure 4:**
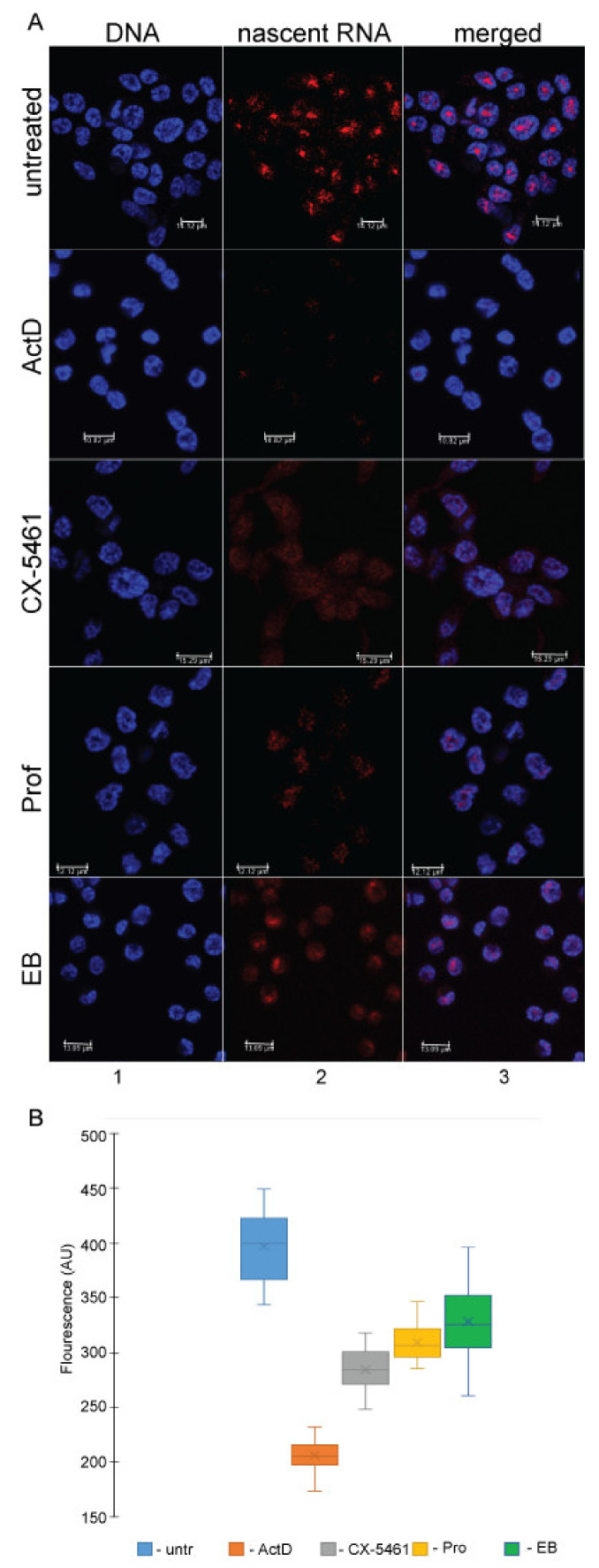
Intercalators preferentially affect Pol I transcription. (**A**) HCC116 p53^−/−^ cells were treated with drug for 30 min or left untreated as indicated. The following concentrations were used: Actinomycin D (ActD): 3 µM; ethidium bromide (EB): 40 µM; proflavine (Pro): 2 µM; CX5461: 5 µM 1 mM 5EU was added to cells and incubation was continued for another hour. Cells were fixed and then permeabilized. Nascent RNA (red, middle panel) was detected using Click-IT^®^ Cell Reaction Buffer Kit (ThermoFisher) supplemented with Cy5 Azide (Sigma-Aldrich). Nucleolar DNA (blue, left panel) was stained with Hoechst 33342. Merged images are shown (right panel). Images were acquired with a Leica TCS SP5 confocal microscope equipped with an × 63 oil-filled objective. (**B**) Box-and-whisker plots of quantification of 5EU incorporation into nascent RNA per cell obtained from experiment shown in (**A**). AU stands for arbitrary units. The median values are shown as horizontal lines.

**Figure 5:**
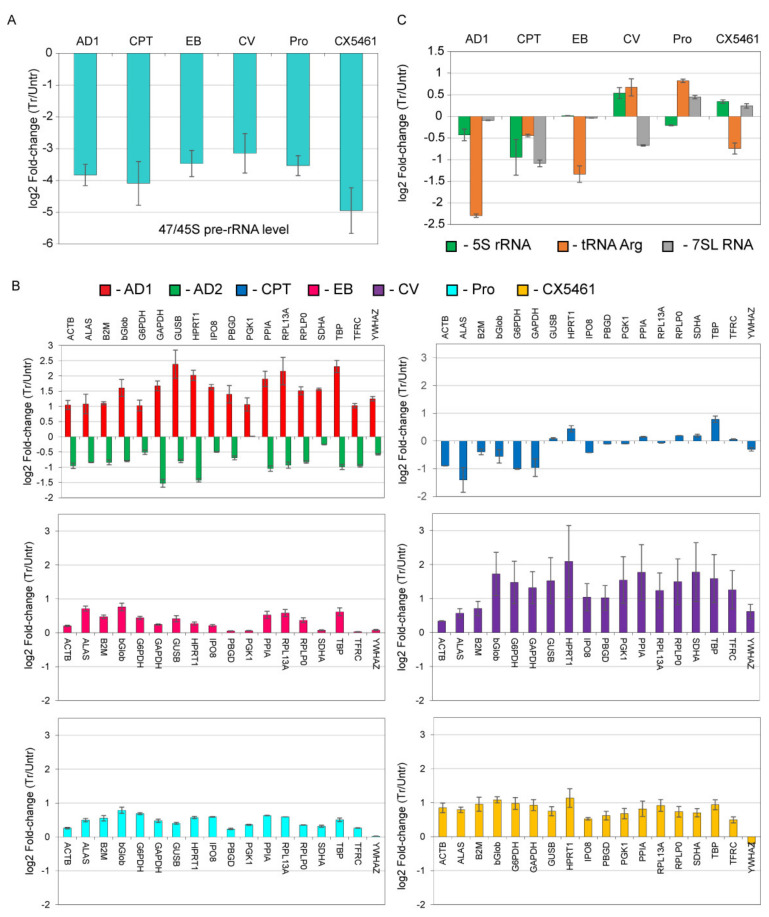
Intercalators have limited effect on general transcription by Pol-II and Pol-III in p53 null background. HCT116 p53^−/−^cells grown to 70% confluence and were incubated with the drugs as indicated for 30 min prior to lysis (AD1—3 nM ActD; AD2—3 µM ActD; CPT—8 µM Camptothecin; EB—40 µM ethidium bromide; CV—20 µM crystal violet; Pro—2 µM proflavine; CX5461—5 µM CX-5461). Total RNA was isolated, reverse transcribed and analyzed by qPCR. (**A**) The combined levels of 47 and 45S pre-rRNA were analyzed in treated and untreated cells using primers described in [17]. The signals were normalized to 18S rRNA and plotted as log2 of treated/untreated ratio. The data represents an average from three independent experiments (*n* = 3); standard deviations are shown. (**B**) The levels of mRNA of 18 genes (as indicated) were analyzed in treated and untreated cells using the Human Housekeeper reference gene plate (Roche). The signals were normalized to 18S rRNA and plotted as log2 of treated/untreated ratio. The data represents an average from three independent experiments (*n* = 3); standard deviations are shown. (**C**) The levels of Pol-III transcripts (as indicated) were analyzed in treated and untreated cells as described in Materials and Methods. The signals were normalized to 18S rRNA and plotted as log2 of treated/untreated ratio. The data represents an average from three independent experiments (*n* = 3); standard deviations are shown.

As expected, a high concentration of ActD (3 μM) almost completely abolished 5EU incorporation and hence RNA synthesis (Figure 4A,B). If the effect of ActD is set to be 100% inhibition, CX-5461 inhibits approximately 60% of all transcription, which is in good agreement with the data suggesting that transcription of Pol-I in actively growing cells can account for up to 70% of total RNA synthesis. Both intercalators have a more moderate inhibitory effect (48% for proflavine and 38% for ethidium bromide), which is consistent with the Pol-I transcription data (Figure 5A).

Importantly, treatment with proflavine and ethidium bromide not only affected the level of nascent RNA synthesis, but also affected the distribution of nascent RNAs. As it is well known, and as shown in Figure 4A, a significant proportion of labelled RNA is localized in the nucleolus of untreated cells. This localization is lost as a result of Pol-I inhibition by CX-5461, and notably as result of intercalator’s treatment, strongly confirming our preliminary conclusion that Pol-I driven transcription is a target for DNA intercalators/grove binders. 

It should be noted that we used relatively short treatment times to pin-point earlier effects, and if treatment time is increased to several hours the inhibitory effect of the intercalators is greatly increased (Supplementary Figure S1).

As a complimentary approach we also measured the effect of intercalators and grove binders on transcription by Pol-II and Pol-III by determining relative expression levels of 18 house-keeping genes (the Human Housekeeper reference gene plate, Roche) and of three Pol-III dependent genes in intercalator treated and untreated HCT116 p53^−/−^ and p53^+/+^ cells as described previously [9].

Compound concentrations in this experiment were sufficient to inhibit about 90% of Pol-I transcription (Figure 5A). We found that, intercalators/groove binders and ActD at 3 nM (AD1) did not repress Pol II/III transcription in p53 null cells (Figure 5B,C). Moreover, all compounds (except CPT) cause an insignificant increase in the transcription of some of the Pol II targets. Importantly, high concentration of ActD causes repression of transcription of all Pol-II targets, therefore serving as a positive inhibitory control. We observed a variable level of inhibitory effect of ActD (AD2) on different targets (as well as different level of stimulatory effect by AD1 and compounds tested) that may be linked to different rate of turnover of particular mRNA, which, as has become evident in recent years, is significantly underestimated especially for many highly transcribed genes in actively growing cells [37,38,39].

In cells expressing wt p53 (Supplementary Figure S2) all compound tested showed an inhibitory effect for the majority of genes tested. This effect was quite significant for our positive control (high concentration of ActD), suggesting that compounds can activate the p53-dependent response, which may affect transcription or/and mRNA turnover. Interestingly, the difference in the response of different genes to the treatment with drugs was also observed in p53 positive cells.

Together, these results indicate that intercalators and groove binders affect Pol-I transcription in cells but have significantly less effect on the transcription by Pol-II and Pol-III, thereby confirming the high sensitivity of Pol-I to compounds that may affect the 3D-structure of DNA.

### 3.4. DNA Intercalators/Groove Binders Affect Recruitment of Pol-I to the rDNA Promoter

**Figure 6:**
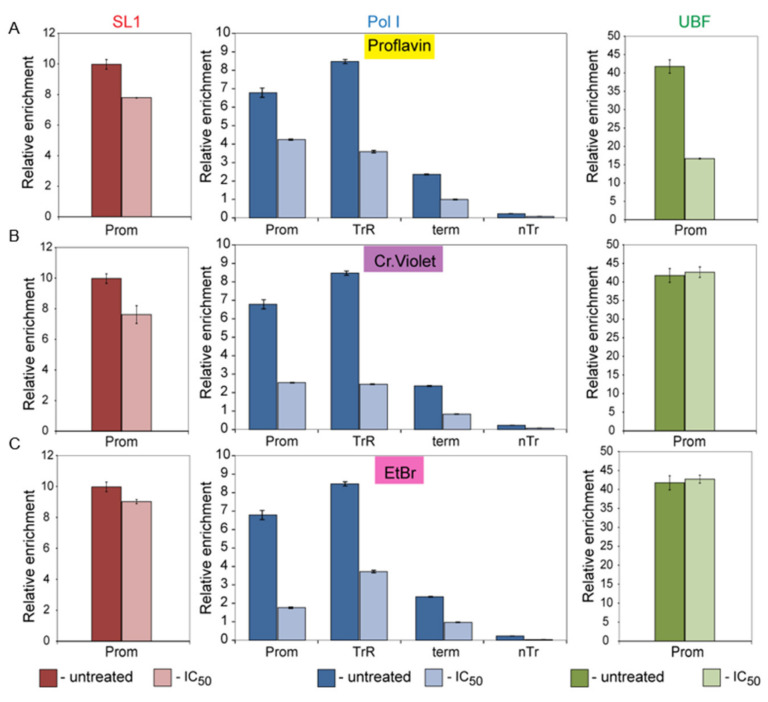
ChIP analysis of the rDNA repeat reveals that intercalating compounds alters Pol-I occupancy at rDNA. HCT116 p53^−/−^ cells grown to 70% confluence and were either with proflavine (Pro) (**A**), or Crystal Violet (CV) (**B**) or Ethidium Bromide (EtB) (**C**) for 30 min prior to chromatin crosslinking. Concentration of all drugs were equal to the IC_50_ *in vivo* (Table 2). ChIP assays were performed using antibodies specific either to TAF_I_110 subunit of human SL1 complex (left panel), or to A194 subunit of human Pol-I complex (middle panel) or to human UBF (right panel). Purified, immunoprecipitated DNA was analyzed by qPCR using specific probes and primers derived from different regions of rDNA repeats as described earlier [18]. Internal standards were used for absolute quantification of immunoprecipitated DNA and chromatin input. The value of each bar represents the difference between the signals from the specific antibody and from the negative control (an appropriate IgG) expressed as % from total chromatin input. Signal representing the transcribed region (TrR) is the average of the combined signal from 5’ETS, 18S, 5.8S and 28S regions of rRNA. Signal representing the non-transcribed region (nTrR) is the average of the combined signal from IGS1 and IGS2 regions. The standard deviations from three independent experiments are shown; *n* = 3.

Our results suggest that the tested intercalators either directly affect binding of Pol-I basal factors to the promoter or affect the tertiary structure of the promoter within the PIC or interfere with the promoter clearance. To clarify the mechanism of action of these drugs we performed chromatin-immunoprecipitation (ChIP) assays using a selection of intercalators from each of the main groups: (GC, AT or general binding; proflavine (Pro), crystal violet (CV) and ethidium bromide (EtBr) respectively) and analyzed the occupancy of basal Pol-I transcription factors (SL1, Pol-I and UBF) at the rDNA before and after treatment at IC_50_ concentration. To exclude any potential effects of p53 activation by these drugs (activation of p53 leads to dissociation of SL1 from the rRNA promoter [40]) we used the HCT116 p53^−/−^ cell line) (Figure 6). 

We found that UBF occupancy was affected by proflavine but not by other drugs. Surprisingly, none of the drugs significantly affected SL1 occupancy at the rRNA promoter in contrast to two other intercalating agents known to affect initiation of transcription (9HE [9] and CX-5461 [8]). Instead, for all drugs tested we observed a dramatic decrease in Pol-I occupancy at the promoter, the transcribed region and the terminator.

### 3.5. Camptothecin Inhibits Initial Stages of Pol-I Transcription Independently from Its Anti-Top1 Activity

**Figure 7:**
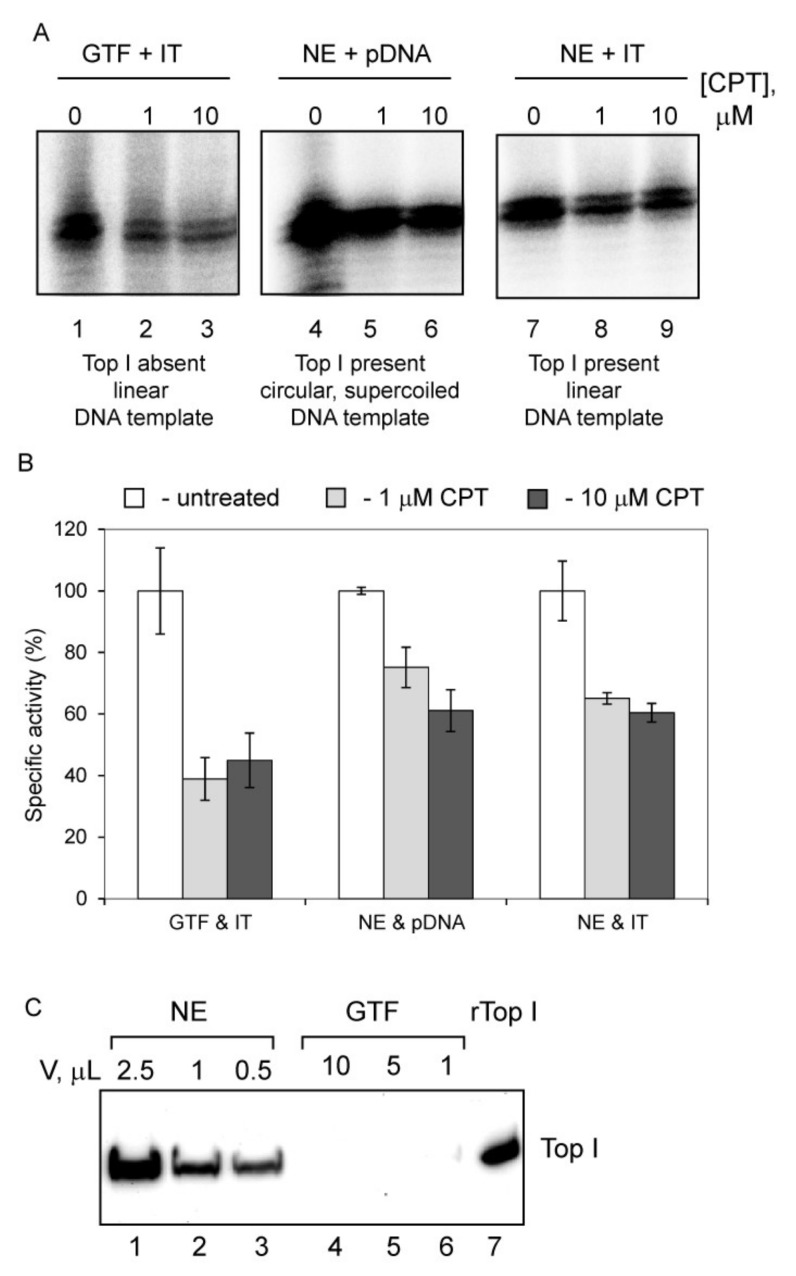
CPT treatment leads to inhibition of Pol-I transcription even in transcription reactions were Top 1 is not present or not required for transcription. (**A**) In vitro transcription reactions were supplemented either with 200 ng immobilized Pol-I promoter (left and right panels) or with 200 ng supercoiled plasmid DNA containing Pol-I promoter (central panel). Reactions were carried out in the presence of either purified GTFs capable of only a single round of transcription and lacking Top 1 (left panel) or HeLa NE capable of multi-round transcription and containing Top 1 (central and right panels). Efficiency of specific transcription was analyzed by S1 Nuclease protection assay and representative images are shown. (**B**) The results were quantified with aid of a phosphoimager. The data expressed as a percentage of the highest value (set at 100%). The data represents an average from three independent experiments (*n* = 3); standard deviations are shown; *p*-values were calculated using one and two-way ANOVA on R software, *p* < 0.05. (**C**) Top 1 is absent in highly purified transcription factors. Various quantities of NE and GTF (as indicated) were analyzed by Western blotting using antibodies against human Top 1.

Camptothecin (CPT) is a compound that inhibits rRNA synthesis in humans by inhibiting Topoisomerase 1 (Top 1) which plays an important role in Pol-I elongation [41,42]. As with many other Topoisomerase inhibitors CPT is a DNA intercalator [43]. In order to compare CPT action with the previously tested intercalators, we investigated the effect of CPT on the Pol-I specific transcription in vitro (Figure 7). In these experiments we used either 440 bp immobilized linear rDNA template (IT) (Figure 7A, lanes 1–3 and 7–9) or supercoiled plasmid DNA (Figure 7A, lanes 4–6). Transcription from the linear template does not require topoisomerase activity because the DNA can freely rotate and no supercoiling can accumulate ahead and behind of the elongating Pol-I. As a control, we used Top I free (Figure 7C, lanes 4–6) reconstituted system containing highly purified general transcription factors (GTFs: Pol Iβ, SL1 and recombinant UBF) in combination with IT (Figure 7A, lanes 1–3). In the other experiments HeLa nuclear extract (NE) that contains a Top I (Figure 7C, lanes 1–3) and capable to support multi-round transcription [14] was supplemented either with circular supercoiled template (efficient transcription may require topoisomerase activity) or with IT (no topoisomerase activity required) (Figure 7A, lanes 4–9). 

Intriguingly, CPT represses Pol-I specific transcription under all conditions (Figure 7A, lanes 1–9) with similar efficiency (Figure 7B), strongly suggesting that Top 1 activity is not required to support transcription from non-chromatinised templates in vitro and that CPT inhibits Pol-I transcription through a Top 1 independent mechanism in a cell-free system. This implies that the Top 1 inhibitory activity of CPT in cells is probably not the only mechanism of its action on Pol I transcription.

## 4. Discussion

The known selective inhibitors of Pol-I transcription are DNA intercalators and we hypothesize that changes in the structure of DNA induced by intercalators (or groove binders) are important for Pol-I inhibiting activity. Our hypothesis is based on work by Denissov and colleagues who investigated the topology of active rDNA repeats [44], our work on ellipticines [9] and advances in the structural biology of Pol-I initiation mechanism [45,46,47,48,49].

In the first study a new core-helix model has been suggested which implies a very important role of 3D-structure of the rDNA transcription unit for the productive synthesis of rRNA. DNA intercalators and groove binders are known to affect the 3D-structure of DNA and we hypothesize that Pol-I transcription would exhibit high sensitivity to these changes induced by such agents, compared to Pol-II and Pol-III (the results that we have observed for ellipticines). By performing in vitro and in cell-based transcription assays, we demonstrate that DNA intercalators and groove binders inhibit Pol-I transcription regardless of their sequence specificity. The tested compounds do not influence the catalytic properties of the enzyme (Figure 2) and have greater inhibitory effect on Pol-I transcription than on Pol-II/III transcription (Figure 3, Figure 4 and Figure 5). On the basis of our results, we cannot rule out that in cells, the tested compounds may have some additional activities leading to rRNA synthesis deregulation, for example affecting rRNA processing. However, we have shown that these compounds are able to inhibit Pol-I specific transcription in a cell-free system with high efficiency. As concluded from elongation complex structures of the three polymerases from yeast [45,46,47,48,49] and Pol II [50] and III [51,52,53,54] from mammals, the general mechanisms of transcript elongation are apparently similar between them, while initiation of Pol-I strongly differs [55]. For these reasons, we can conclude that early events in the Pol-I transcription cycle are most likely targets for DNA intercalators and groove binders both in vitro and in cells. 

Supported by in vivo occupancy studies (Figure 6), our results suggest that intercalators/groove binders may target the rDNA promoter and affect either PIC formation or promoter clearance. However, the exact mechanism of inhibition awaits further investigation. Potentially, such agents can interfere either with (i) binding of basal factors to the rRNA promoter (similar to the ellipticines and CX-5461) or (ii) with ternary structure of the rRNA promoter. 

The promoter consists of three key elements: upstream binding site (UCE), core region and linker region [56,57]. During PIC formation the promoter undergoes conformational changes which lead to spatial rearrangement of its elements, resulting in the binding of Pol-I transcription factor UBF to the UCE, and SL1 to the core element. As a result, the core and UCE become spatially close which allows SL1 and UBF to interact. Moreover, it is likely that both factors will have additional interactions with DNA outside their “normal” binding sites. This newly established network of interactions stabilizes both SL1 and UBF at the promoter and increase the efficiency of Pol-I recruitment as well as promoter clearance [58,59]. Importantly, the linker region is not involved in the specific protein-DNA interactions, but its length is critical for formation of a fully competent PIC at the rDNA promoter [57,60].

Furthermore, structural analysis of the SL1-homologous ‘Core Factor’ (CF) and an initially transcribing Pol I complex in the yeast system [47,48,49]) underlines that the 3D arrangement of initiation factors binding upstream of the transcription start site (TSS) is crucial for efficient initiation and may be conserved throughout organisms as suggested earlier [61]. Specifically, a characteristic bend between proximal and distal rDNA promoter elements is required to allow the establishment of contacts between (i) Pol I and proximal promoter elements, (ii) CF and distal promoter elements and (iii) the conserved CF/SL1-subunit Rrn11/TAF1A with the Pol I protrusion domain. This analysis led to the hypothesis that a combination of biophysical promoter DNA features, i.e., bendability and meltability, defines the rDNA promoter throughout organisms, rationalizing why promoter-opening and initial transcription do not require additional energy in the Pol-I system [49]. Thus, any alteration of promoter DNA properties by intercalators/groove binders may have drastic effects on the cooperation of initiation factors or the productive recruitment initiation-competent Pol-I/Rrn3 complex [62,63], [64]. In addition, Rrn3 is similar in yeast and human [65] and required for SL1-dependent initiation in the human transcription system [13], and a similar DNA architecture was predicted in the yeast and human rRNA promoters, suggesting a mechanistic conservation of the Pol I initiation system [49]. This idea is supported by the sensitivity of Pol-I transcription in evolutionarily distinct organisms, which have significant differences in the organization of the Pol-I transcriptional apparatus, to the same Pol-I inhibitors (e.g., human, yeast [9], Plasmodium (KP, unpublished data) and Trypanosome [66] are all sensitive to 9HE and CX-5461). 

## 5. Conclusions

Thus, we propose that the three-dimensional architecture of the rDNA promoter within the pre-initiation complex plays an important and previously not fully recognized role in the sensitivity of rDNA transcription to various chemical agents and, in particular, to DNA intercalators and groove binders. This can have certain implications for our under-standing of the sensitivity of cells to environmental contamination.

One of the unexpected results of this study is the finding of Top 1 independent inhib-itory effect of Camptothecin (CPT). Our data strongly suggests that CPT repress Pol-I tran-scription by not only inhibiting Top 1 and affecting Pol-I elongation, but also by interfering with the initial stages of transcription via a Top 1 independent mechanism, and this was not previously recognized.

## Figures and Tables

**Table 1:** In vitro IC_50_ concentrations for a range of DNA intercalators/groove binders.

Compound	DNA Binding Specificity	K_D_, M^−1^	Ref.	* Pol I IC_50_, M	±SD, M
Ethidium bromide	non-specific intercalator	1.0 × 10^7^	[21]	6.18 × 10^−8^	0.06 × 10^−8^
Proflavine	GC intercalator	8.4 × 10^6^	[22]	3.50 × 10^−7^	0.14 ×10^−7^
9HE	GC intercalator	5.0 × 10^7^	[23]	4.20 × 10^−7^	1.5 × 10^−7^
Hoescht 33258	AT (minor groove)	3.0 × 10^9^	[24]	6.59 × 10^−7^	0.24 × 10^−7^
DAPI	AT (minor groove) GC intercalator	3.0 × 10^8^ 1.2 × 10^5^	[25] [26]	6.60 × 10^−7^	0.14 × 10^−7^
Propidium Iodide	non-specific intercalator	1.0 × 10^8^	[21]	7.06 × 10^−7^	0.28 × 10^−7^
Acridine Orange	GC intercalator	5.0 × 10^4^	[27]	1.55 × 10^−6^	0.20 × 10^−6^
Thionine	GC intercalator	1.4 × 10^5^	[28]	4.70 × 10^−6^	0.31 × 10^−6^
Crystal violet	AT (major groove)	6.0 × 10^3^	[29]	3.46 × 10^−5^	0.52 × 10^−5^
Methyl green	AT (major groove)	2.0 × 10^4^	[30]	5.3 × 10^−3^	0.3 × 10^−3^

* IC_50_ concentrations were determined in vitro, in reactions supplemented with 1 µL of HeLa nuclear extract and 100 ng of plasmid DNA containing rRNA promoter. rRNA transcripts were detected by S1 nuclease protection assay in triplicates as described in Methods. IC_50_ and standard deviation was calculated using GraphPad Prism (version 5.0) software.

**Table 2:** In vivo IC_50_ concentrations for a selection of DNA intercalators/groove binders.

Compound	DNA Binding Specificity	* IC_50_, M (rRNA Synthesis)	±SD, M
Ethidium bromide	non-specific intercalator	9.16 × 10^−6^	0.33 × 10^−6^
Proflavine	GC	4.76 × 10^−7^	0.27 × 10^−7^
Camptothecin	GC	1.99 × 10^−6^	0.18 × 10^−6^
9HE	GC	1.5 × 10^−7^	0.50 × 10^−7^
DAPI	AT (minor groove)	3.2 × 10^−1^	0.11 × 10^−1^
Crystal violet	AT (major groove)	4.94 × 10^−6^	0.67 × 10^−6^

*—IC_50_ concentrations were determined in vivo using p53 null HCT116 cells in triplicates. Total RNA was analyzed by S1 nuclease protection assay as described in Methods. IC_50_ and standard deviation was calculated using GraphPad Prism (version 5.0) software.

## Supplementary Materials

The following are available online at https://www.mdpi.com/article/10.3390/genes12091412/s1, Figure S1. Longer treatment with intercalators resulted in higher negative effect on the nascent RNA synthesis; Figure S2. Intercalators have moderate negative effect on Pol-II transcription in the presence of wt p53; Table S1: Supplementary Table S1. Antibody used in this work; Table S2: Supplementary Table S2. Sequences of all primers and probes used in this work; Table S3: In vitro IC_50_ concentrations for CX-5461 and CPT; Supplementary Data S1: Sequence of pcfTAF110 plasmid; Supplementary Data S2: Sequence of CMV-Fr DNA fragment.
