# Supplementary material for: DNA Intercalators Inhibit Eukaryotic Ribosomal RNA Synthesis by Impairing the Initiation of Transcription
Source: Genes (Basel). 2021 Sep 14;12(9):1412. doi: 10.3390/genes12091412 (PMC8466728; doi:10.3390/genes12091412)
Supplement: Supplementary file 1 [file genes-12-01412-s001.zip › genes-1250091-supplementary.pdf]

Supplementary data

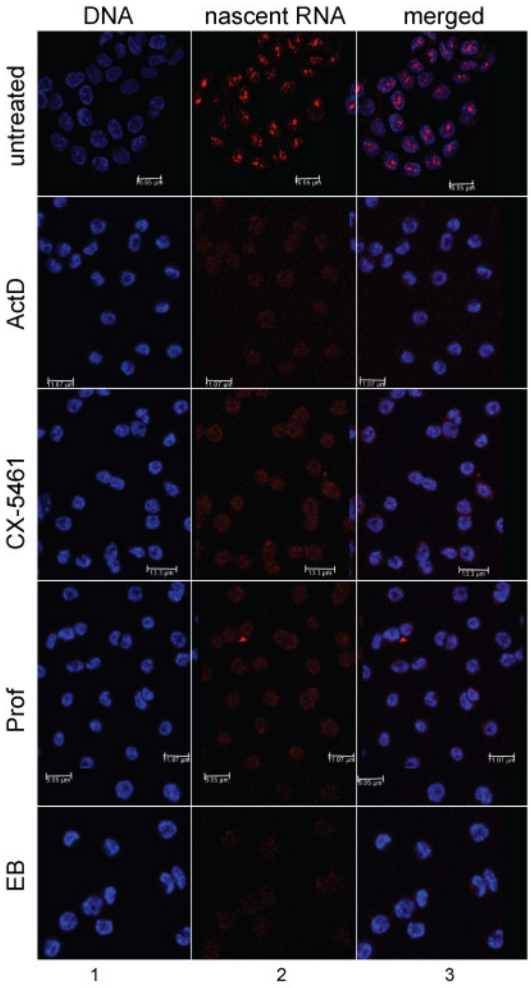

Figure S1. Long treatment with intercalators affects all transcription activity in cells. HCT116 p53$^{-/-}$ cells were treated with drug for 4 hours or left untreated as indicated. Following concentrations were used: Actinomycin D (ActD): 3 µM; ethidium bromide (EB): 40 µM; Proflavine (Pro): 2 µM; CX5461: 5 µM 1 mM 5EU was added to cells and incubation was continued for another hour. Cells were fixed and then permeabilized. Nascent RNA (red, middle panel) was detected using Click-IT® Cell Reaction Buffer Kit (ThermoFisher) supplemented with Cy5 Azide (Sigma). Nucleolar DNA (blue, left panel) was stained with Hoechst 33342. Merged images are shown (right panel). Images were acquired with a Leica TCS SP5 confocal microscope equipped with an x63 oil-filled objective.

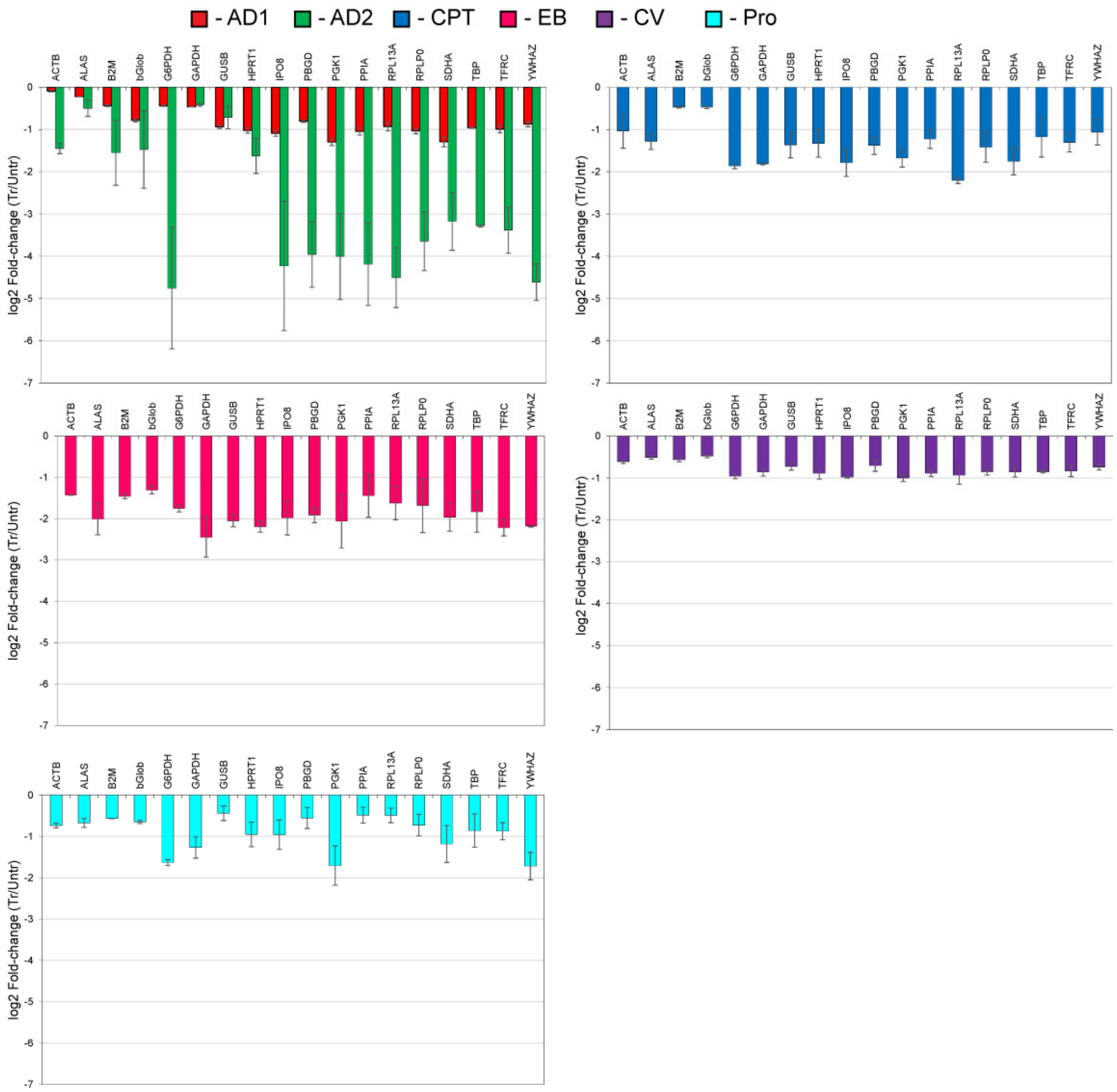

Figure S2. Intercalators have moderate negative effect on Pol-II transcription in the presence of wt p53. HCT116 p53$^{+/+}$ cells grown to 70% confluence and were incubated with the drugs as indicated for 30 minutes prior to lysis (AD1 – 3 nM Actinomycin D; AD2 – 3 µM Actinomycin D; CPT – 8 µM Camptothecin; EB – 40 µM ethidium bromide; CV – 20 µM crystal violet; Pro - 2 µM Proflavine; CX5461 - 5 µM CX-5461). Total RNA was isolated, reverse transcribed and analysed by qPCR. The levels of mRNA of 18 genes (as indicated) were analysed in treated and untreated cells using the Human Housekeeper reference gene plate (Roche). The signals were normalised to 18S rRNA and plotted as log2 of treated/untreated ratio. The data represents an average from three independent experiments (n=3); standard deviations are shown.

Supplementary Table S1. Antibody used in this work

Antibody	Origin	Company	Cat.number	WB	ChIP/IP
RPA135 (N-17)	Goat	Santa Cruz	Sc-17913		12.5µg
TAF_I63	Rabbit	JZ lab	N/A		10 µl
UBF (H300)	Rabbit	Santa Cruz	Sc-9131		12.5µg
Topo I (H-300)	Rabbit	Santa Cruz	Sc-10783	1:1000	
Rabbit IgG	Rabbit	Sigma	I5006		12.5µg
Sheep IgG	Sheep	Sigma	I5131		12.5µg

Supplementary Table S2. Sequences of all primers and probes used in this work

Template	Primer/oligo	Sequence 5'-3'	Modification	Amplicon/probe
plasmid pcfTAF110	template_Pol_II_F	GAAAAATAAACAAATAGGGGTTCC	5′ biotin	PCR product; 5'-biotynilated CMV_FR, 1354 bp
	template_Pol_II_R	TGCTCAGTCACATCCAGCAC	-	
CMV_FR	Pol_IITr _R	CAGTCACATCCAGCACGACT	-	PCR product 412 bp
	Pol_IITr_F	GGAGACCCAAGCTGGCTAGT	-	
	Pol_IITr2_F	CCCCTTCACCATGGATTACA	-	PCR product 127 bp
	Pol_IITr2_R	CCAGCTGCACATGAAAGAGA	-	
	Pol-II_Pr	ACGACGATAAGGACTTCCCC	5'-HEX, 3'-BHQ2	qPCR dual labelled probe

Reference genes panel

Position	Transcript Identifier	Assay ID	Forward Sequenz	Forward Sequenz	Reverse Sequenz	Reverse Sequenz	Kontrolle	Referenz Gen	Design Info.
A1	NR_003286	104092	GACGGACCAGAGCGAAAG	18	CGTCTTCGAACCTCCGACT	19	0	1	NR_003286//EntrezGene//RN18S1
B1	ENST00000349264	102065	CCGTGTGAACCATGTGACTTT	21	CCTCCATGATGCTGCTTACA	20	0	1	ENST00000349264//Ensembl//B2M
C1	ENST00000229239	143641	TGGTATCGTGGAAGGACTCA	20	GCAGGGATGATGTTCTGGAG	20	0	1	ENST00000229239//Ensembl//GAPDH
D1	ENST00000256079	102132	GTGTACACACTGGCAGAGCAC	21	GCCTCCCTGTTGTTCAATCT	20	0	1	ENST00000256079//Ensembl//IPO8
E1	ENST00000355968	102088	TTCATCTGCACTGCCAAGAC	20	CACTTTGCCAAACACCACAT	20	0	1	ENST00000355968//Ensembl//PPIA
F1	ENST00000264932	145170	TCCACTACATGACGGAGCAG	20	CCATCTTCAGTTCTGCTAAACG	22	0	1	ENST00000264932//Ensembl//SDHA
G1	ENST00000353245	102125	GATCCCCAATGCTTCACAAG	20	TGCTTGTTGTGACTGATCGAC	21	0	1	ENST00000353245//Ensembl//YWHAZ
H1	ENST00000322644	102977	TCCGTATTCGCATCATGAAC	20	TCATCCATCTTGTCCACCAC	20	1	0	ENST00000322644//Ensembl//POLR2A
A2	ENST00000331789	101125	GGCCAGGTCATCACCATT	18	GGATGCCACAGGACTCCAT	19	0	1	ENST00000331789//Ensembl//ACTB

B2		102141	TGCAGGCTGCCTATCAGAA	19	GCGAGCTTAGTGATACTTGTGG	22	0	1	ENST00000335295//Ensembl//HBB
C2	ENST00000304895	102074	CGCCCTGCCTATCTGTATTC	20	TCCCCACAGGGAGTGTGTAG	20	0	1	ENST00000304895//Ensembl//GUSB
D2	ENST00000278715	102110	TGCCCTGGAGAAGAATGAAG	20	CAGCATCATGAGGGTTTTCC	20	0	1	ENST00000278715//Ensembl//HMBS
E2	ENST00000391857	102119	CTGGACCGTCTCAAGGTGTT	20	GCCCCAGATAGGCAAACTT	19	0	1	ENST00000391857//Ensembl//RPL13A
F2	ENST00000392092	101145	GAACATCATGGATCAGAACAACA	23	ATAGGGATTCCGGGAGTCAT	20	0	1	ENST00000392092//Ensembl//TBP
G2	ENST00000322644	102978	CCTGAGTCCGGATGAACTG	19	GCCTCCCTCAGTCGTCTCT	19	1	0	ENST00000322644//Ensembl//POLR2A
H2	ENST00000360110	102422	TGGGTTTTTGTTACCTTTATGGTT	24	GGAGGTAACATGCAAATAATGTGA	24	1	0	ENST00000360110//Ensembl//TFRC
A3	ENST00000394965	102108	GAAATGAATGCCGTGAGGAA	20	CCTCCATCGGTTTTCACACT	20	0	1	ENST00000394965//Ensembl//ALAS1
B3	ENST00000393564	102098	TCCATCAGTCGGATACACACA	21	CACCAGATGGTGGGGTAGAT	20	0	1	ENST00000393564//Ensembl//G6PD
C3	ENST00000370796	102079	TGACCTTGATTTATTTTGCATACC	24	CGAGCAAGACGTTCAGTCCT	20	0	1	ENST00000370796//Ensembl//HPRT1
D3	ENST00000373316	102083	GGAGAACCTCCGCTTTCAT	19	GCTGGCTCGGCTTTAACC	18	0	1	ENST00000373316//Ensembl//PGK1
E3	ENST00000228306	101144	TCGACAATGGCAGCATCTAC	20	GCCAATCTGCAGACAGACAC	20	0	1	ENST00000228306//Ensembl//RPLP0
F3	ENST00000392396	102095	TTGAGAAAACAATGCAAAATGTG	23	CCCAGTTGCTGTCCTGATATAGA	23	0	1	ENST00000392396//Ensembl//TFRC
G3	ENST00000322644	102127	GCAAATTCACCAAGAGAGACG	21	CACGTCGACAGGAACATCAG	20	1	0	ENST00000322644//Ensembl//POLR2A
H3	ENST00000360110	107847	TGGGTTTTTGTTACCTTTATGGTT	24	GGAGGTAACATGCAAATAATGTGA	24	1	0	ENST00000360110//Ensembl//TFRC
A4	NR_003286	104092	GACGGACCAGAGCGAAAG	18	CGTCTTCGAACCTCCGACT	19	0	1	NR_003286//EntrezGene//RN18S1
B4	ENST00000349264	102065	CCGTGTGAACCATGTGACTTT	21	CCTCCATGATGCTGCTTACA	20	0	1	ENST00000349264//Ensembl//B2M
C4	ENST00000229239	143641	TGGTATCGTGGAAGGACTCA	20	GCAGGGATGATGTTCTGGAG	20	0	1	ENST00000229239//Ensembl//GAPDH
D4	ENST00000256079	102132	GTGTACACACTGGCAGAGCAC	21	GCCTCCCTGTTGTTCAATCT	20	0	1	ENST00000256079//Ensembl//IPO8
E4	ENST00000355968	102088	TTCATCTGCACTGCCAAGAC	20	CACTTTGCCAAACACCACAT	20	0	1	ENST00000355968//Ensembl//PPIA

F4	ENST000 00264932	145 170	TCCACTACATGAC GGAGCAG	20	CCATCTTCAGTTCT GCTAAACG	22	0	1	ENST00000264932// Ensembl//SDHA
G4	ENST000 00353245	102 125	GATCCCCAATGCT TCACAAG	20	TGCTTGTTGTGAC TGATCGAC	21	0	1	ENST00000353245// Ensembl//YWHAZ
H4	ENST000 00322644	102 977	TCCGTATTCGCAT CATGAAC	20	TCATCCATCTTGTC CACCAC	20	1	0	ENST00000322644// Ensembl//POLR2A
A5	ENST000 00331789	101 125	GGCCAGGTCATC ACCATT	18	GGATGCCACAGGA CTCCAT	19	0	1	ENST00000331789// Ensembl//ACTB
B5		102 141	TGCAGGCTGCCTA TCAGAA	19	GCGAGCTTAGTGA TACTTGTGG	22	0	1	ENST00000335295// Ensembl//HBB
C5	ENST000 00304895	102 074	CGCCCTGCCTATC TGTATTC	20	TCCCCACAGGGAG TGTGTAG	20	0	1	ENST00000304895// Ensembl//GUSB
D5	ENST000 00278715	102 110	TGCCCTGGAGAA GAATGAAG	20	CAGCATCATGAGG GTTTTCC	20	0	1	ENST00000278715// Ensembl//HMBS
E5	ENST000 00391857	102 119	CTGGACCGTCTCA AGGTGTT	20	GCCCCAGATAGGC AAACTT	19	0	1	ENST00000391857// Ensembl//RPL13A
F5	ENST000 00392092	101 145	GAACATCATGGA TCAGAACAACA	23	ATAGGGATTCCGG GAGTCAT	20	0	1	ENST00000392092// Ensembl//TBP
G5	ENST000 00322644	102 978	CCTGAGTCCGGAT GAACTG	19	GCCTCCCTCAGTC GTCTCT	19	1	0	ENST00000322644// Ensembl//POLR2A
H5	ENST000 00360110	102 422	TGGGTTTTTGTTA CCTTTATGGTT	24	GGAGGTAACATGC AAATAATGTGA	24	1	0	ENST00000360110// Ensembl//TFRC
A6	ENST000 00394965	102 108	GAAATGAATGCC GTGAGGAA	20	CCTCCATCGGTTTT CACACT	20	0	1	ENST00000394965// Ensembl//ALAS1
B6	ENST000 00393564	102 098	TCCATCAGTCGGA TACACACA	21	CACCAGATGGTGG GGTAGAT	20	0	1	ENST00000393564// Ensembl//G6PD
C6	ENST000 00370796	102 079	TGACCTTGATTTA TTTTGCATACC	24	CGAGCAAGACGTT CAGTCCT	20	0	1	ENST00000370796// Ensembl//HPRT1
D6	ENST000 00373316	102 083	GGAGAACCTCCG CTTTCAT	19	GCTGGCTCGGCTT TAACC	18	0	1	ENST00000373316// Ensembl//PGK1
E6	ENST000 00228306	101 144	TCGACAATGGCA GCATCTAC	20	GCCAATCTGCAGA CAGACAC	20	0	1	ENST00000228306// Ensembl//RPLP0
F6	ENST000 00392396	102 095	TTGAGAAAACAA TGCAAAATGTG	23	CCCAGTTGCTGTC CTGATATAGA	23	0	1	ENST00000392396// Ensembl//TFRC
G6	ENST000 00322644	102 127	GCAAATTCACCA AGAGAGACG	21	CACGTCGACAGGA ACATCAG	20	1	0	ENST00000322644// Ensembl//POLR2A
H6	ENST000 00360110	107 847	TGGGTTTTTGTTA CCTTTATGGTT	24	GGAGGTAACATGC AAATAATGTGA	24	1	0	ENST00000360110// Ensembl//TFRC
A7	NR_00328 6	104 092	GACGGACCAGAG CGAAAG	18	CGTCTTCGAACCT CCGACT	19	0	1	NR_003286//EntrezG ene//RN18S1

B7	ENST00000349264	102065	CCGTGTGAACCATGTGACTTT	21	CCTCCATGATGCTGCTTACA	20	0	1	ENST00000349264//Ensembl//B2M
C7	ENST00000229239	143641	TGGTATCGTGGAAGGACTCA	20	GCAGGGATGATGTTCTGGAG	20	0	1	ENST00000229239//Ensembl//GAPDH
D7	ENST00000256079	102132	GTGTACACACTGGCAGAGCAC	21	GCCTCCCTGTTGTTCAATCT	20	0	1	ENST00000256079//Ensembl//IPO8
E7	ENST00000355968	102088	TTCATCTGCACTGCCAAGAC	20	CACTTTGCCAAACACCACAT	20	0	1	ENST00000355968//Ensembl//PPIA
F7	ENST00000264932	145170	TCCACTACATGACGGAGCAG	20	CCATCTTCAGTTCTGCTAAACG	22	0	1	ENST00000264932//Ensembl//SDHA
G7	ENST00000353245	102125	GATCCCCAATGCTTCACAAG	20	TGCTTGTTGTGACTGATCGAC	21	0	1	ENST00000353245//Ensembl//YWHAZ
H7	ENST00000322644	102977	TCCGTATTCGCATCATGAAC	20	TCATCCATCTTGTCCACCAC	20	1	0	ENST00000322644//Ensembl//POLR2A
A8	ENST00000331789	101125	GGCCAGGTCATCACCATT	18	GGATGCCACAGGACTCCAT	19	0	1	ENST00000331789//Ensembl//ACTB
B8		102141	TGCAGGCTGCCTATCAGAA	19	GCGAGCTTAGTGATACTTGTGG	22	0	1	ENST00000335295//Ensembl//HBB
C8	ENST00000304895	102074	CGCCCTGCCTATCTGTATTC	20	TCCCCACAGGGAGTGTGTAG	20	0	1	ENST00000304895//Ensembl//GUSB
D8	ENST00000278715	102110	TGCCCTGGAGAAGAATGAAG	20	CAGCATCATGAGGGTTTTCC	20	0	1	ENST00000278715//Ensembl//HMBS
E8	ENST00000391857	102119	CTGGACCGTCTCAAGGTGTT	20	GCCCCAGATAGGCAAACTT	19	0	1	ENST00000391857//Ensembl//RPL13A
F8	ENST00000392092	101145	GAACATCATGGATCAGAACAACA	23	ATAGGGATTCCGGGAGTCAT	20	0	1	ENST00000392092//Ensembl//TBP
G8	ENST00000322644	102978	CCTGAGTCCGGATGAACTG	19	GCCTCCCTCAGTCGTCTCT	19	1	0	ENST00000322644//Ensembl//POLR2A
H8	ENST00000360110	102422	TGGGTTTTTGTTACCTTTATGGTT	24	GGAGGTAACATGCAAATAATGTGA	24	1	0	ENST00000360110//Ensembl//TFRC
A9	ENST00000394965	102108	GAAATGAATGCCGTGAGGAA	20	CCTCCATCGGTTTTCACACT	20	0	1	ENST00000394965//Ensembl//ALAS1
B9	ENST00000393564	102098	TCCATCAGTCGGATACACACA	21	CACCAGATGGTGGGGTAGAT	20	0	1	ENST00000393564//Ensembl//G6PD
C9	ENST00000370796	102079	TGACCTTGATTTATTTTGCATACC	24	CGAGCAAGACGTTCAGTCCT	20	0	1	ENST00000370796//Ensembl//HPRT1
D9	ENST00000373316	102083	GGAGAACCTCCGCTTTCAT	19	GCTGGCTCGGCTTTAACC	18	0	1	ENST00000373316//Ensembl//PGK1
E9	ENST00000228306	101144	TCGACAATGGCAGCATCTAC	20	GCCAATCTGCAGACAGACAC	20	0	1	ENST00000228306//Ensembl//RPLP0

F9	ENST00000392396	102095	TTGAGAAAACAATGCAAAATGTG	23	CCCAGTTGCTGTCCTGATATAGA	23	0	1	ENST00000392396//Ensembl//TFRC
G9	ENST00000322644	102127	GCAAATTCACCAAGAGAGACG	21	CACGTCGACAGGAACATCAG	20	1	0	ENST00000322644//Ensembl//POLR2A
H9	ENST00000360110	107847	TGGGTTTTTGTTACCTTTATGGTT	24	GGAGGTAACATGCAAATAATGTGA	24	1	0	ENST00000360110//Ensembl//TFRC
A10	NR_003286	104092	GACGGACCAGAGCGAAAG	18	CGTCTTCGAACCTCCGACT	19	0	1	NR_003286//EntrezGene//RN18S1
B10	ENST00000349264	102065	CCGTGTGAACCATGTGACTTT	21	CCTCCATGATGCTGCTTACA	20	0	1	ENST00000349264//Ensembl//B2M
C10	ENST00000229239	143641	TGGTATCGTGGAAGGACTCA	20	GCAGGGATGATGTTCTGGAG	20	0	1	ENST00000229239//Ensembl//GAPDH
D10	ENST00000256079	102132	GTGTACACACTGGCAGAGCAC	21	GCCTCCCTGTTGTTCAATCT	20	0	1	ENST00000256079//Ensembl//IPO8
E10	ENST00000355968	102088	TTCATCTGCACTGCCAAGAC	20	CACTTTGCCAAACACCACAT	20	0	1	ENST00000355968//Ensembl//PPIA
F10	ENST00000264932	145170	TCCACTACATGACGGAGCAG	20	CCATCTTCAGTTCTGCTAAACG	22	0	1	ENST00000264932//Ensembl//SDHA
G10	ENST00000353245	102125	GATCCCCAATGCTTCACAAG	20	TGCTTGTTGTGACTGATCGAC	21	0	1	ENST00000353245//Ensembl//YWHAZ
H10	ENST00000322644	102977	TCCGTATTCGCATCATGAAC	20	TCATCCATCTTGTCCACCAC	20	1	0	ENST00000322644//Ensembl//POLR2A
A11	ENST00000331789	101125	GGCCAGGTCATCACCATT	18	GGATGCCACAGGACTCCAT	19	0	1	ENST00000331789//Ensembl//ACTB
B11		102141	TGCAGGCTGCCTATCAGAA	19	GCGAGCTTAGTGATACTTGTGG	22	0	1	ENST00000335295//Ensembl//HBB
C11	ENST00000304895	102074	CGCCCTGCCTATCTGTATTC	20	TCCCCACAGGGAGTGTGTAG	20	0	1	ENST00000304895//Ensembl//GUSB
D11	ENST00000278715	102110	TGCCCTGGAGAAGAATGAAG	20	CAGCATCATGAGGGTTTTCC	20	0	1	ENST00000278715//Ensembl//HMBS
E11	ENST00000391857	102119	CTGGACCGTCTCAAGGTGTT	20	GCCCCAGATAGGCAAACTT	19	0	1	ENST00000391857//Ensembl//RPL13A
F11	ENST00000392092	101145	GAACATCATGGATCAGAACAACA	23	ATAGGGATTCCGGGAGTCAT	20	0	1	ENST00000392092//Ensembl//TBP
G11	ENST00000322644	102978	CCTGAGTCCGGATGAACTG	19	GCCTCCCTCAGTCGTCTCT	19	1	0	ENST00000322644//Ensembl//POLR2A
H11	ENST00000360110	102422	TGGGTTTTTGTTACCTTTATGGTT	24	GGAGGTAACATGCAAATAATGTGA	24	1	0	ENST00000360110//Ensembl//TFRC
A12	ENST00000394965	102108	GAAATGAATGCCGTGAGGAA	20	CCTCCATCGGTTTTCACACT	20	0	1	ENST00000394965//Ensembl//ALAS1

B12	ENST000 00393564	102 098	TCCATCAGTCGGA TACACACA	21	CACCAGATGGTGG GGTAGAT	20	0	1	ENST00000393564// Ensembl//G6PD
C12	ENST000 00370796	102 079	TGACCTTGATTTA TTTTGCATACC	24	CGAGCAAGACGTT CAGTCCT	20	0	1	ENST00000370796// Ensembl//HPRT1
D12	ENST000 00373316	102 083	GGAGAACCTCCG CTTTCAT	19	GCTGGCTCGGCTT TAACC	18	0	1	ENST00000373316// Ensembl//PGK1
E12	ENST000 00228306	101 144	TCGACAATGGCA GCATCTAC	20	GCCAATCTGCAGA CAGACAC	20	0	1	ENST00000228306// Ensembl//RPLP0
F12	ENST000 00392396	102 095	TTGAGAAAACAA TGCAAAATGTG	23	CCCAGTTGCTGTC CTGATATAGA	23	0	1	ENST00000392396// Ensembl//TFRC
G12	ENST000 00322644	102 127	GCAAATTCACCA AGAGAGACG	21	CACGTCGACAGGA ACATCAG	20	1	0	ENST00000322644// Ensembl//POLR2A
H12	ENST000 00360110	107 847	TGGGTTTTTGTTA CCTTTATGGTT	24	GGAGGTAACATGC AAATAATGTGA	24	1	0	ENST00000360110// Ensembl//TFRC

Supplementary Table S3. ***In vitro*** **IC_{50} concentrations for CX-5461 and CPT**

Compound	*Pol I IC_{50}, M	±SD, M
CX-5461	$1.21x10^{-7}$	$0.22x10^{-7}$
CPT	$1.10x10^{-6}$	$0.08x10^{-6}$

* IC_{50} concentrations were determined in vitro, in reactions supplemented with 1 µl of HeLa nuclear extract and 100 ng of plasmid DNA containing rRNA promoter. rRNA transcripts were detected by S1 nuclease protection assay in triplicates as described in Methods. IC50 and standard deviation was calculated using GraphPad Prism (version 9.11) software.

Supplementary Data 1. Sequence of pcfTAF110.

cttgtacaaagtggttgatctagagggcccgcggttcgaaggtaagcctatccctaaccctctcctcggtctcgattctacgcgtacc
ggttagtaatgagtttaaacgggggaggctaactgaaacacggaaggagacaataccggaaggaacccgcgctatgacggc
aataaaaagacagaataaaacgcacgggtgttgggtcgtttgttcataaacgcggggttcggtcccagggctggcactctgtcg
ataccccaccgagaccccattggggccaatacgcccgcgtttcttccttttccccaccccaccccccaagttcgggtgaaggccc
agggctcgcagccaacgtcggggcggcaggccctgccatagcagatctgcgcagctggggctctagggggtatccccacgcg
ccctgtagcggcgcattaagcgcggcgggtgtggtggttacgcgcagcgtgaccgctacacttgccagcgccctagcgcccgct
cctttcgctttcttcccttcctttctcgccacgttcgccggctttccccgtcaagctctaaatcggggcatccctttagggttccgatttagt
gctttacggcacctcgaccccaaaaaacttgattagggtgatggttcacgtagtgggccatcgccctgatagacggtttttcgccctt
tgacgttggagtccacgttctttaatagtggactcttgttccaaactggaacaacactcaaccctatctcggtctattcttttgatttataa
gggattttggggatttcggcctattggttaaaaaatgagctgatttaacaaaaatttaacgcgaattaattctgtggaatgtgtgtcagt
tagggtgtggaaagtccccaggctccccagcaggcagaagtatgcaaagcatgcatctcaattagtcagcaaccaggtgtgga
aagtccccaggctccccagcaggcagaagtatgcaaagcatgcatctcaattagtcagcaaccatagtcccgcccctaactcc
gcccatcccgcccctaactccgcccagttccgcccattctccgccccatggctgactaattttttttatttatgcagaggccgaggcc
gcctctgcctctgagctattccagaagtagtgaggaggcttttttggaggcctaggcttttgcaaaaagctcccgggagcttgtatat
ccattttcggatctgatcaagagacaggatgaggatcgtttcgcatgattgaacaagatggattgcacgcaggttctccggccgctt
gggtggagaggctattcggctatgactgggcacaacagacaatcggctgctctgatgccgccgtgttccggctgtcagcgcagg
ggcgcccggttctttttgtcaagaccgacctgtccggtgccctgaatgaactgcaggacgaggcagcgcggctatcgtggctggc
cacgacgggcgttccttgcgcagctgtgctcgacgttgtcactgaagcgggaagggactggctgctattgggcgaagtgccggg
gcaggatctcctgtcatctcaccttgctcctgccgagaaagtatccatcatggctgatgcaatgcggcggctgcatacgcttgatcc
ggctacctgcccattcgaccaccaagcgaaacatcgcatcgagcgagcacgtactcggatggaagccggtcttgtcgatcagg

atgatctggacgaagagcatcaggggctcgcgccagccgaactgttcgccaggctcaaggcgcgcatgcccgacggcgagg
atctcgtcgtgacccatggcgatgcctgcttgccgaatatcatggtggaaaatggccgcttttctggattcatcgactgtggccggct
gggtgtggcggaccgctatcaggacatagcgttggctacccgtgatattgctgaagagcttggcggcgaatgggctgaccgcttc
ctcgtgctttacggtatcgccgctcccgattcgcagcgcatcgccttctatcgccttcttgacgagttcttctgagcgggactctggggt
tcgcgaaatgaccgaccaagcgacgcccaacctgccatcacgagatttcgattccaccgccgccttctatgaaaggttgggcttc
ggaatcgttttccgggacgccggctggatgatcctccagcgcggggatctcatgctggagttcttcgcccaccccaacttgtttattg
cagcttataatggttacaaataaagcaatagcatcacaaatttcacaaataaagcatttttttcactgcattctagttgtggtttgtcca
aactcatcaatgtatcttatcatgtctgtataccgtcgacctctagctagagcttggcgtaatcatggtcatagctgtttcctgtgtgaaa
ttgttatccgctcacaattccacacaacatacgagccggaagcataaagtgtaaagcctggggtgcctaatgagtgagctaactc
acattaattgcgttgcgctcactgcccgctttccagtcgggaaacctgtcgtgccagctgcattaatgaatcggccaacgcgcggg
gagaggcggtttgcgtattgggcgctcttccgcttcctcgctcactgactcgctgcgctcggtcgttcggctgcggcgagcggtatca
gctcactcaaaggcggtaatacggttatccacagaatcaggggataacgcaggaaagaacatgtgagcaaaaggccagca
aaaggccaggaaccgtaaaaaggccgcgttgctggcgtttttccataggctccgcccccctgacgagcatcacaaaaatcgac
gctcaagtcagaggtggcgaaacccgacaggactataaagataccaggcgtttccccctggaagctccctcgtgcgctctcctgt
tccgaccctgccgcttaccggatacctgtccgcctttctcccttcgggaagcgtggcgctttctcaatgctcacgctgtaggtatctca
gttcggtgtaggtcgttcgctccaagctgggctgtgtgcacgaaccccccgttcagcccgaccgctgcgccttatccggtaactatc
gtcttgagtccaacccggtaagacacgacttatcgccactggcagcagccactggtaacaggattagcagagcgaggtatgta
ggcggtgctacagagttcttgaagtggtggcctaactacggctacactagaaggacagtatttggtatctgcgctctgctgaagcc
agttaccttcggaaaaagagttggtagctcttgatccggcaaacaaaccaccgctggtagcggtggtttttttgtttgcaagcagca
gattacgcgcagaaaaaaaggatctcaagaagatcctttgatcttttctacggggtctgacgctcagtggaacgaaaactcacgtt
aagggattttggtcatgagattatcaaaaaggatcttcacctagatccttttaaattaaaaatgaagttttaaatcaatctaaagtatat
atgagtaaacttggtctgacagttaccaatgcttaatcagtgaggcacctatctcagcgatctgtctatttcgttcatccatagttgcct
gactccccgtcgtgtagataactacgatacgggagggcttaccatctggccccagtgctgcaatgataccgcgagacccacgct
caccggctccagatttatcagcaataaaccagccagccggaagggccgagcgcagaagtggtcctgcaactttatccgcctcc
atccagtctattaattgttgccgggaagctagagtaagtagttcgccagttaatagtttgcgcaacgttgttgccattgctacaggcat
cgtggtgtcacgctcgtcgtttggtatggcttcattcagctccggttcccaacgatcaaggcgagttacatgatcccccatgttgtgca
aaaaagcggttagctccttcggtcctccgatcgttgtcagaagtaagttggccgcagtgttatcactcatggttatggcagcactgc
ataattctcttactgtcatgccatccgtaagatgcttttctgtgactggtgagtactcaaccaagtcattctgagaatagtgtatgcggc
gaccgagttgctcttgcccggcgtcaatacgggataataccgcgccacatagcagaactttaaaagtgctcatcattggaaaac
gttcttcggggcgaaaactctcaaggatcttaccgctgttgagatccagttcgatgtaacccactcgtgcacccaactgatcttcag
catcttttactttcaccagcgtttctgggtgagcaaaaacaggaaggcaaaatgccgcaaaaaagggaataagggcgacacg
gaaatgttgaatactcatactcttcctttttcaatattattgaagcatttatcagggttattgtctcatgagcggatacatatttgaatgtattt
agaaaaataaacaaataggggttccgcgcacatttccccgaaaagtgccacctgacgtcgacggatcgggagatctcccgatc
ccctatggtcgactctcagtacaatctgctctgatgccgcatagttaagccagtatctgctccctgcttgtgtgttggaggtcgctgagt
agtgcgcgagcaaaatttaagctacaacaaggcaaggcttgaccgacaattgcatgaagaatctgcttagggttaggcgttttgc
gctgcttcgcgatgtacgggccagatatacgcgttgacattgattattgactagttattaatagtaatcaattacggggtcattagttca
tagcccatatatggagttccgcgttacataacttacggtaaatggcccgcctggctgaccgcccaacgacccccgcccattgacg
tcaataatgacgtatgttcccatagtaacgccaatagggactttccattgacgtcaatgggtggactatttacggtaaactgcccact
tggcagtacatcaagtgtatcatatgccaagtacgccccctattgacgtcaatgacggtaaatggcccgcctggcattatgcccag
tacatgaccttatgggactttcctacttggcagtacatctacgtattagtcatcgctattaccatggtgatgcggttttggcagtacatca
atgggcgtggatagcggtttgactcacggggatttccaagtctccaccccattgacgtcaatgggagtttgttttggcaccaaaatc
aacgggactttccaaaatgtcgtaacaactccgccccattgacgcaaatgggcggtaggcgtgtacggtgggaggtctatataa
gcagagctctctggctaactagagaacccactgcttactggcttatcgaaattaatacgactcactatagggagacccaagctgg
ctagttaagctatcaacaagtttgtacaaaaaagcaggctccgcggccgccccccttcaccatggattacaaggatgacgacgat
aaggacttccccagctccctccgccctgcgttgtttctgaccggccccttggtctgagcgacgtccctgacctctctttcatgtgcag
ctggcgagacgcactgactctgccagaggcccagccccagaactcagagaatggggcactgcatgtgaccaaggacctgct
gtgggagccggcaacccctgggcctctccccatgctgcctcccctcatcgatccctgggaccctggcctgactgcccgggacctg
cttttccgcggagggtaccggtatcggaagcggccccgagtcgtgctggatgtgactgagcagatcagccggttcctcttggatca

tggagacgtagcctttgcgcccctggggaagctgatgctggagaatttcaagctggagggagcggggagccgcactaagaag
aagacagtggtcagtgtgaagaagctgctccaggacctcggtggacaccagccctgggggtgtccctgggcttacctcagcaa
ccgacagcgccgcttctctatcctcgggggccccatcctgggcacgtcggtggcgagccacttggcagagctgctgcacgagg
agctggtgctgcggtgggagcagctgcttctggatgaggcctgcactgggggcgcgctggcctgggttcctggaaggacacccc
agttcgggcagctggtctaccctgctggaggcgcccaggacaggctgcatttccaagaggtcgttctgaccccaggtgacaatc
cccaattccttgggaaacctggacgcatccagctccagggacctgtccggcaagtggtgacatgcaccgtccagggagaaagt
aaggcccttatatacactttcctccctcactggctgacctgctacctgacccctggccctttccatccctcctcagctctgctggccgtc
cgctctgactaccactgtgccgtgtggaagtttggtaaacagtggcagccaacccttctgcaggcgatgcaggtggagaaaggg
gccacggggatcagcctcagccctcacctgcccggggagctggccatctgcagccgctcgggagccgtctgcctgtggagccc
tgaggatgggctgcggcaaatctacagggaccctgagaccctcgtgttccgggactcctcttcgtggcgttgggcagacttcactg
cgcaccctcgggtgctgaccgtgggtgaccgcaccggagtgaagatgctggacactcagggcccgccgggctgtggtctgttg
ctttttcgtttgggggcagaggcttcgtgccagaaaggggaacgtgtcctgcttacccagtacctggggcactccagccccaaatg
cctccccccctactcttcatctcgtctgtacccagttctctctctacctagtggacgagcgccttcccctggtgccgatgctgaagtgga
accatggcctcccctccccgctcctgctggcccgactgctgcctccgccccggcccagctgcgtgcagcccctgctcctcggagg
ccagggtgggcagctgcagctgctgcacctggcaggagaaggggcgtcggtgccccgcctggcaggcccccccccagtctctt
ccttccaggatcgactccctccctgcatttcctctgctggagcctaagatccagtggcggctgcaggagcgcctgaaagcaccga
ccataggtctggctgccgtcgtcccgcccttgccctcagcgcccacaccaggcctggtgctcttccagctctcggcggcgggaga
tgtcttctaccagcagctccgcccccaggtggactccagcctccgcagagatgctgggcctcctggcgacacccaacctgactg
ccatgcccccacagcttcctggacctcccaggacactgccggctgcagccagtggctgaaggccctgctaaaagtgcccctgg
ctcctcctgtgtggacagcacccaccttcacccaccgccagatgctgggcagcacagagctgcggagggaggaagaggaag
ggcagcggctgggtgtgctccgcaaggccatggcccgagggcagctcctgctgcagagagacctgggctccctccctgcggc
agagccaccccctgcacccgagtcaggcctagaggacaagctcagtgagcgcctgggggaagcctgggcaggccgaggg
gctgcctggtgggagaggcagcagggcaggacctcggagcccgggagacagaccaggcggcccaagcgccggacccag
ctgtccagcagcttttcgctcagtggccatgtggatccgtcagaggacaccagctcccctcatagccctgagtggccacctgctga
tgctctgcccctgccccccacgaccccgccctcccaggagttgactccggatgcatgcgcccagggcgtcccatcagagcagc
ggcagatgctccgtgactacatggccaagctaccaccccagagggacaccccaggctgtgccaccacacctccccactccca
ggcctccagcgtccgggccactcgctcccagcagcacacacccgtcctctctagctctcagcccctccggaagaagcctcgaat
gggcttctgaaagggtgggcgcgccgacccagcttt

Supplementary Data 2. Sequence of CMV_Fr.

gaaaaataaacaaataggggttccgcgcacatttccccgaaaagtgccacctgacgtcgacggatcgggagatctcccgatcc
cctatggtcgactctcagtacaatctgctctgatgccgcatagttaagccagtatctgctccctgcttgtgtgttggaggtcgctgagta
gtgcgcgagcaaaatttaagctacaacaaggcaaggcttgaccgacaattgcatgaagaatctgcttagggttaggcgttttgcg
ctgcttcgcgatgtacgggccagatatacgcgttgacattgattattgactagttattaatagtaatcaattacggggtcattagttcat
agcccatatatggagttccgcgttacataacttacggtaaatggcccgcctggctgaccgcccaacgacccccgcccattgacgt
caataatgacgtatgttcccatagtaacgccaatagggactttccattgacgtcaatgggtggactatttacggtaaactgcccactt
ggcagtacatcaagtgtatcatatgccaagtacgccccctattgacgtcaatgacggtaaatggcccgcctggcattatgcccagt
acatgaccttatgggactttcctacttggcagtacatctacgtattagtcatcgctattaccatggtgatgcggttttggcagtacatca
atgggcgtggatagcggtttgactcacggggatttccaagtctccaccccattgacgtcaatgggagtttgttttggcaccaaaatc
aacgggactttccaaaatgtcgtaacaactccgccccattgacgcaaatgggcggtaggcgtgtacggtgggaggtctatataa
gcagagctctctggctaactagagaacccactgcttactggcttatcgaaattaatacgactcactatagggagacccaagctgg
ctagttaagctatcaacaagtttgtacaaaaaagcaggctccgcggccgccccccttcaccatggattacaaggatgacgacgat
aaggacttccccagctccctccgccctgcgttgtttctgaccggccccccttggtctgagcgacgtccctgacctctctttcatgtgcag
ctggcgagacgcactgactctgccagaggcccagccccagaactcagagaatggggcactgcatgtgaccaaggacctgct
gtgggagccggcaaccccctgggcctctccccatgctgcctcccctcatcgatccctgggaccctggcctgactgcccgggacctg
cttttccgcggagggtaccggtatcggaagcggccccgagtcgtgctggatgtgactgagca
